# Mesoporous NH_4_NiPO_4_·H_2_O for High-Performance Flexible All-Solid-State Asymmetric Supercapacitors

**DOI:** 10.3389/fchem.2019.00118

**Published:** 2019-03-07

**Authors:** Yong Liu, Xiaoliang Zhai, Keke Yang, Fei Wang, Huijie Wei, Wanhong Zhang, Fengzhang Ren, Huan Pang

**Affiliations:** ^1^Collaborative Innovation Center of Nonferrous Metals of Henan Province, Henan Key Laboratory of High-Temperature Structural and Functional Materials, School of Materials Science and Engineering, Henan University of Science and Technology, Luoyang, China; ^2^Henan Key Laboratory of Non-Ferrous Materials Science & Processing Technology, Henan University of Science and Technology, Luoyang, China; ^3^School of Chemistry and Chemical Engineering, Yangzhou University, Yangzhou, China

**Keywords:** NH_4_NiPO_4_·H_2_O, flexible supercapacitor, asymmetrical, all-solid-state, electrochemical performances

## Abstract

Nowadays, wearable energy storage devices have been growing rapidly, but flexible systems with both excellent cycling stability and decent flexibility are still challenging. In this work, a flexible all-solid-state NH_4_NiPO_4_·H_2_O//graphene supercapacitor with remarkable performance was successfully assembled. When cycled at a current density of 5 mA cm^−2^, the device delivered 121 mF cm^−2^, and showed good cycling stability after 3,000 cycles. Moreover, the all-solid-state NH_4_NiPO_4_·H_2_O//graphene supercapacitor also exhibit high mechanical flexibility with well-maintained specific capacitance, even under bending to arbitrary angles (up to 180°) and different weights (up to 50 g).

## Introduction

Recently, for the reason of environmental and energy issues, research on energy storage have become one of hot spots all over the world (Wang et al., 2017, 2019; Zheng et al., 2017; Huang et al., 2018a,b,c; Liu et al., 2018; Wang F. et al., 2018; Wang H. et al., 2018; Wang H. K. et al., 2018; Zhang et al., 2018a; Zhao et al., 2018). Among the energy storage systems, supercapacitors have sparked increasing attention due to their high power density and long cycle life (Conway, 1999; Aricò et al., 2005; Miller and Simon, 2008; Yuan et al., 2012; Bin Jiang et al., 2018; Zhu et al., 2018). One type of supercapacitors, pseudocapacitors, which include fast Faradic reactions on the electrodes, could deliver greater specific capacitance than electrochemical double-layer capacitors, which could make the device have higher energy density (Dai et al., 2018; Gao et al., 2018; Zhang et al., 2018b; Zhao et al., 2018a; Zheng et al., 2018). For the electrode materials of pseudocapacitors, electrochemical active materials are often used, such as transition-metal oxides [e.g., RuO_2_ (Zhai et al., 2018), NiO (Zuo et al., 2016), MnO_2_ (Yang et al., 2016), Co_3_O_4_ (Zhang et al., 2016a)], and conducting polymers (Xie and Wang, 2016). Nevertheless, the high price of Ruthenium makes it hardly be utilized as electrode materials for pseudocapacitors. In this case, it is crucial to synthesize an electrode material with low cost and high performance.

On the other hand, portable devices generally require small size, light weight, which the traditional capacitors could not achieve, and all of these limit the development of this area (Huang et al., 2018). As a newly developed energy-storage device, the flexible all-solid-state supercapacitors are small and light compared to the conventional capacitors (Lv et al., 2018). And flexible all-solid-state supercapacitor could deliver much higher energy density than conventional capacitors (Gao et al., 2014a; Wei et al., 2015; Yousaf et al., 2016; Zhang et al., 2016b; Wu et al., 2018). Furthermore, with two electrodes made of different materials, these asymmetric supercapacitors could show better performance in energy density. Together with their high power density, flexible asymmetric all-solid-state supercapacitor are promising for the wearable energy storage systems (Zhang et al., 2016b; Wu et al., 2018).

In the past few years, ammonium/transition metal phosphate NH_4_MPO_4_**·**H_2_O (M = Co^2+^, Ni^2+^) have been studied as electrodes in the field of supercapacitors (Pang et al., 2012; Zhao et al., 2013; Wang et al., 2014a). For instance, Wang and his colleagues utilized a facile hydrothermal method to synthesize layered NH_4_CoPO_4_**·**H_2_O microbundles which consist of 1D layered microrods (Wang et al., 2014a). The layered microbundle electrode showed good high-rate capability as well as excellent cycling stability. In the previous work, we have successful fabricated mesoporous NH_4_NiPO_4_**·**H_2_O nanostructures using one-pot hydrothermal method (Zhao et al., 2013). In this work, we assembled them into flexible all-solid-state asymmetric supercapacitors and studied their electrochemical performances. The specific capacitance of the device can reach 121 mF cm^−2^, and shows good long-term cycling stability. And this device exhibit excellent mechanical flexibility under bending to arbitrary angles (up to 180°) and different weights (even 50 g).

## Materials and Methods

### Synthesis of Mesoporous NH_4_NiPO_4_·H_2_O Nanostructures

NH_4_NiPO_4_·H_2_O nanostructures were synthesized by reacting 0.40 g Ni(NO_3_)_2_ and 0.40 g (NH_4_)_3_PO_4_ at 200°C for 45 h under hydrothermal condition in 20.0 mL ethylene glycol, and the autoclave was then cooled to room temperature as described elsewhere (Zhao et al., 2013). The green and yellow precipitates were obtained and filtered. After being washed with distilled water and ethanol repeatly, the final product was obtained after being dried in air for 24 h.

### Characterizations

The crystal structures of the samples were analyzed by X-ray diffraction (XRD) (Rigaku-Ultima III with Cu *K*α radiation, λ = 1.5418 Å). The microstructures of as-prepared samples were revealed using a field-emission scanning electron microscope (FESEM; JEOL JSM-6701F, 5.0 kV), transmission electron microscopy (TEM) and high-resolution transmission electron microscopy (HRTEM) (JEM-2100, 200 kV). Nitrogen adsorption–desorption isotherms were measured on a Gemini VII 2390 Analyzer at 77 K, and the specific surface area was calculated by the Brunauer-Emmett-Teller (BET) method.

### Fabrication of Flexible All-Solid-State NH_4_NiPO_4_·H_2_O//Graphene Supercapacitors

The PET substrates were first deposited with a layer of Pt film (about 3–5 nm thick) and then coated with the slurry containing the active materials (NH_4_NiPO_4_·H_2_O or graphene via a procedure similar to that in the three-electrode system and were used as the working electrode after drying). Meanwhile, 1.52 g PVA was mixed into 10 ml deionized water to form a mixture. After the mixture is clarified at the constant temperature of 75°C,the prepared 5 ml 3 mol/L KOH are slowly dripped into the mixture with continuous stirring. Then the gel-like electrolyte was obtained. Then, two pieces of such electrodes were immersed in the gel solution for 5–10 min to coat a layer of gel electrolyte. After the excess water was vaporized, two pieces of such electrodes containing electrolyte were pressed together with sandwiched structures. Finally, the stacked all-solid-state NH_4_NiPO_4_·H_2_O//graphene asymmetric supercapacitors were fabricated.

### Electrochemical Measurements

Electrochemical study on all-solid-state NH_4_NiPO_4_·H_2_O//graphene asymmetric supercapacitor was carried out using an electrochemical working-station (CHI 660D, Shanghai Chenhua). The electrochemical performance measurements were conducted in a conventional two-electrode system with graphene electrode as counter and reference electrode. Cyclic voltammetry (CV) and galvanostatic charge-discharge methods were used to investigate capacitive properties of all-solid-state NH_4_NiPO_4_·H_2_O//graphene asymmetric supercapacitor with a potential window between 0 and 1.4 V. And electrochemical impedance spectroscopy (ESI) measurements were carried out by using PARSTAT2273 at 0.4 V over the frequency range of 100 kHz to 10 mHz with an amplitude of 5 mV.

## Results and Discussions

**Figure 1:**
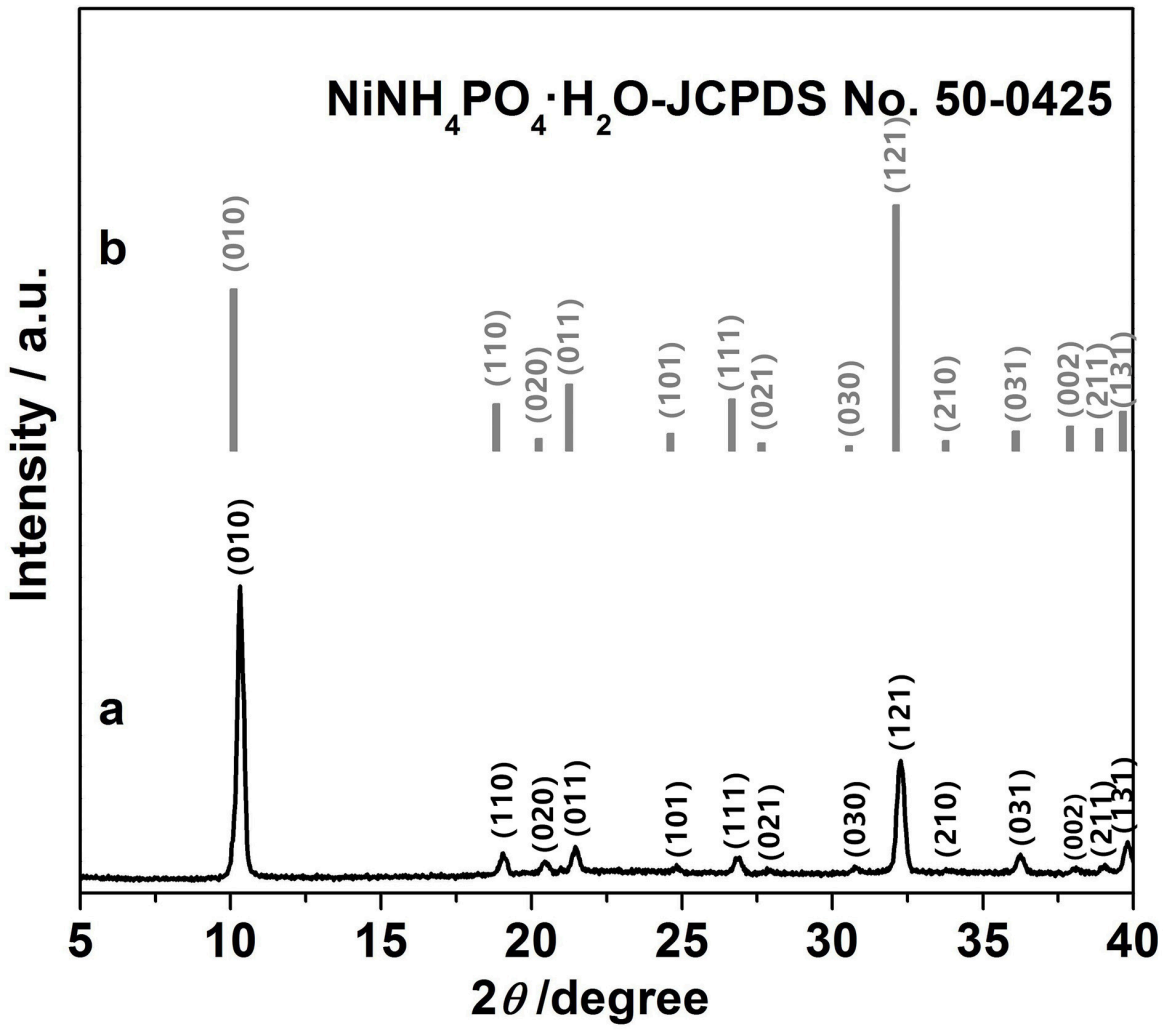
**(a)** X-ray diffraction patterns of as-prepared NH_4_NiPO_4_·H_2_O samples and **(b)** corresponding JCPDS No. 50-0425.

As shown in Figure 1b, all peaks correspond well to those of NH_4_NiPO_4_·H_2_O (JCPD No. 50-0425, Figure 1a), indicating the good crystallinity of the samples. This is consistent with the observations reported elsewhere (Zhao et al., 2013). Figure S1b shows the typical N_2_ adsorption-desorption isotherms of mesoporous NH_4_NiPO_4_·H_2_O, and from the calculation, the specific surface area of the sample was ~418 m^2^ g^−1^ and pore-sizes were within the range about 2.0–18.5 nm (Figure S1a). The presence of mesoporous provides channels for the ion transport, and high specific surface area could facilitate the contact of electrolyte and electrode, which are beneficial to the electrochemical properties of electrode.

**Figure 2:**
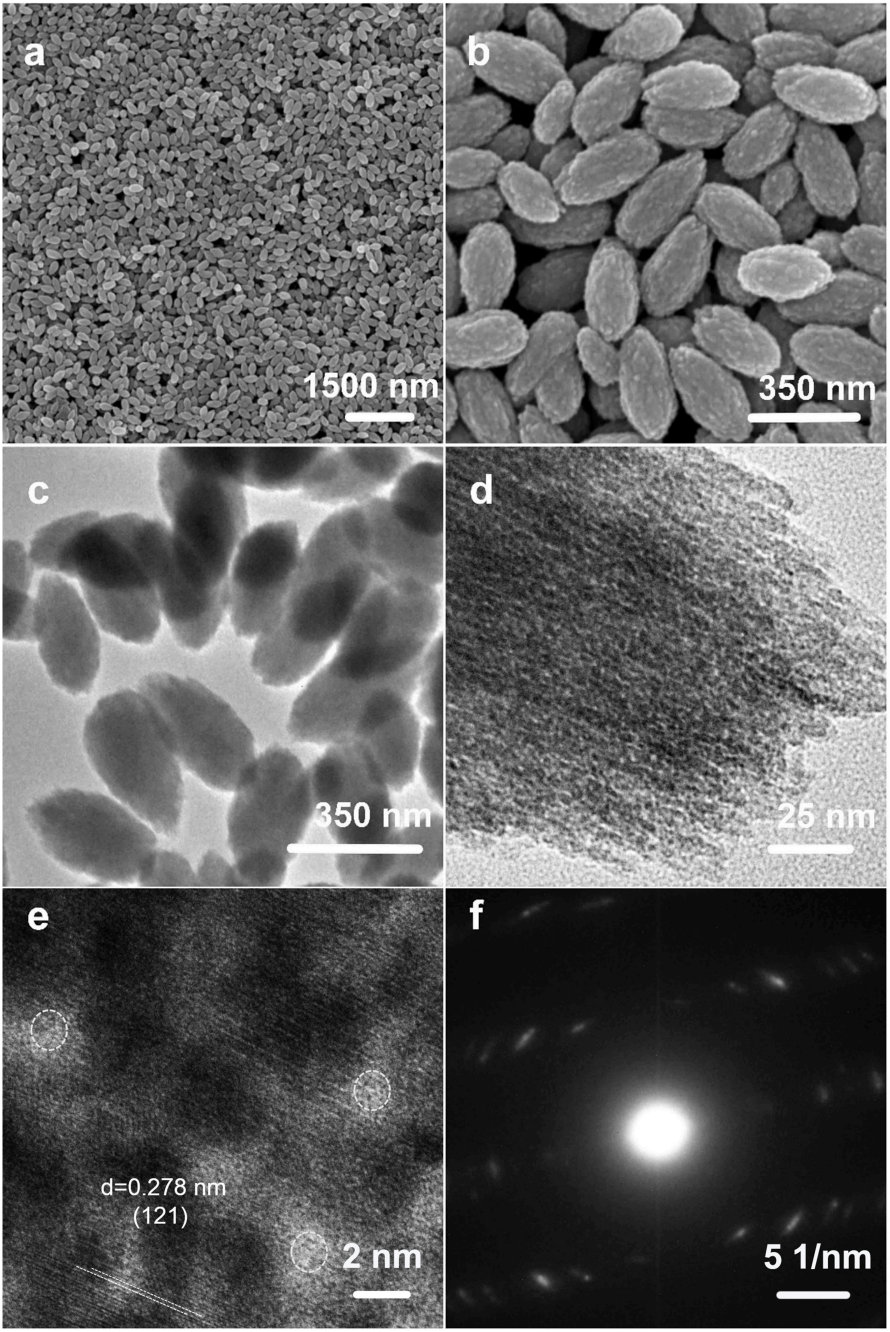
**(a,b)** SEM images, **(c,d)** TEM images, **(e)** HRTEM image, and **(f)** selected area electron diffraction patterns of as-prepared NH_4_NiPO_4_·H_2_O samples.

The morphologies of the as-prepared NH_4_NiPO_4_·H_2_O samples were examined by FESEM and TEM. As shown in Figures 2a,b, the samples are in uniform nano-almond structures, and even when the scale bar is 1.5 μm (Figure 2a), showing the high uniformity of nanostructures. The sizes of single nano-almond are in the range of 300~350 nm. Furthermore, the uniform shape and size were further proved by TEM, which are shown in Figures 2c,d. Figures 2e,f show the HRTEM and selected area electron diffraction (SAED) pattern of as-prepared NH_4_NiPO_4_·H_2_O samples. The d-spacing of lattice fringes in Figure 2e is ~0.278 nm, which is corresponding to the (121) lattice spacing of NH_4_NiPO_4_·H_2_O. The SAED patterns in Figure 2f confirm the polycrystalline nature of the samples, which show NH_4_NiPO_4_·H_2_O phase. As shown in Figure 2e, the measured diameters of pores are ~2.0 nm, and the porous structure may facilitate electrolyte access, resulting in fast ion intercalation and extraction.

**Figure 3:**
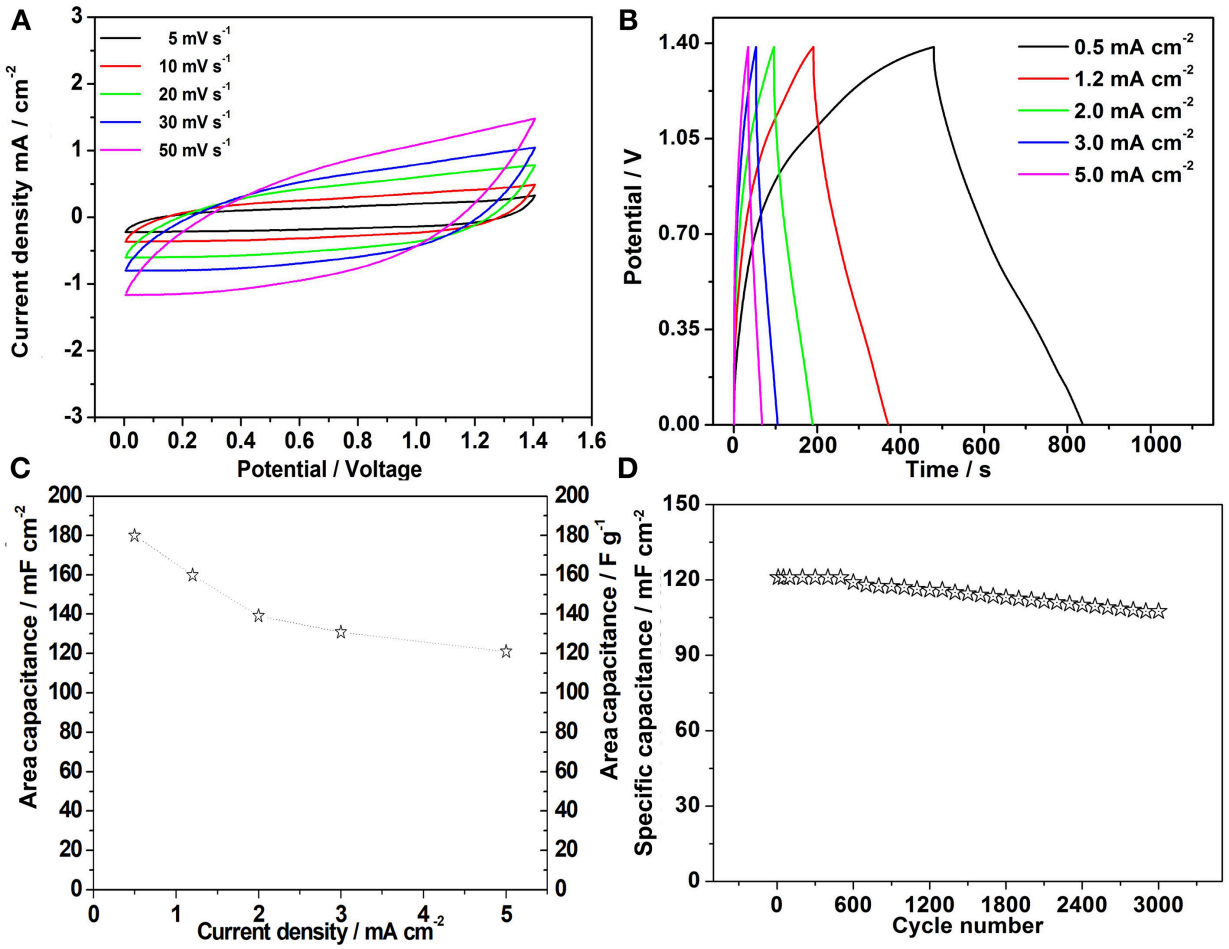
**(A)** Cyclic Voltammetry curves of the hybrid supercapacitor in different scan rates; **(B)** Galvanostatic charge-discharge curves with different current densities; **(C)** Specific capacitance with different current densities; **(D)** Charge-discharge cycling test at a current density of 5.0 mA cm^−2^.

In this work, flexible all-solid-state hybrid supercapacitors were assembled using as-prepared NH_4_NiPO_4_·H_2_O and graphene as positive and negative electrode, respectively. The CV and galvanostatic charge and discharge tests were carried out to test the electrochemical properties of the samples. As the Figure 3A shows, the charging voltage of the device is 0 to 1.4V. When the scan rates range from 5 to 50 mV s^−1^. The curves show a quasi-rectangular geometry, which shows that the sample not only has the characteristic of pseudo capacitance, but also has the characteristics of electric double layer capacitance at these rates (Gao et al., 2014b). Furthermore, when rate is as high as 50 mV s^−1^, the shape of CV curve could still preserved, indicating that the hybrid supercapacitor has very good rate capability (Dai et al., 2018). When the hybrid supercapacitor was charged and discharged in the current density of 0.2, 0.5, 1.2, 2.0, 3.0, 5.0 mA cm^−2^, as the Figure 3B shows, these curves are approximately in triangular shape, which means the supercapacitor have excellent reversibility and capacitance at each current density. And the capacitances is calculated from galvanostatic charge-discharge curves by the following Formula:

(1) $$\begin{array}{r} C_{\text{spec}}=\left(\text{I}\times \Delta \text{t}\right)/\left(\Delta \text{V}\times S\right) \end{array}$$

Where *I* is the current density, *t* is the discharge time, *V* is the potential range (*V* = 1.4 V) and *S* is the area of the supercapacitors (Roldán et al., 2015). After calculation, we plotted the specific capacitance of the supercapacitor. As shown in Figure 3C. When the current density is 0.5 mA cm^−2^, its areal specific capacitances could achieve 180 mF cm^−2^. Remarkably, even at as high as 5 mA cm^−2^, this value can still reach 121 mF cm^−2^. The capacity retention rate is about 88.8%, after 3,000 cycles with the current density of 5 mA cm^−2^ (Figure 3D). This capacitance decay may be attributed to some irreversible reactions between the electrodes and electrolyte (Wang et al., 2014b). Noticeably, even after 3,000 cycles, the nanostructured morphology of the electrode material was well-sustained (Figure S2). A comparison of the electrochemical performance of the supercapacitors with other hybrid solid state devices are shown in Table S1.

**Figure 4:**
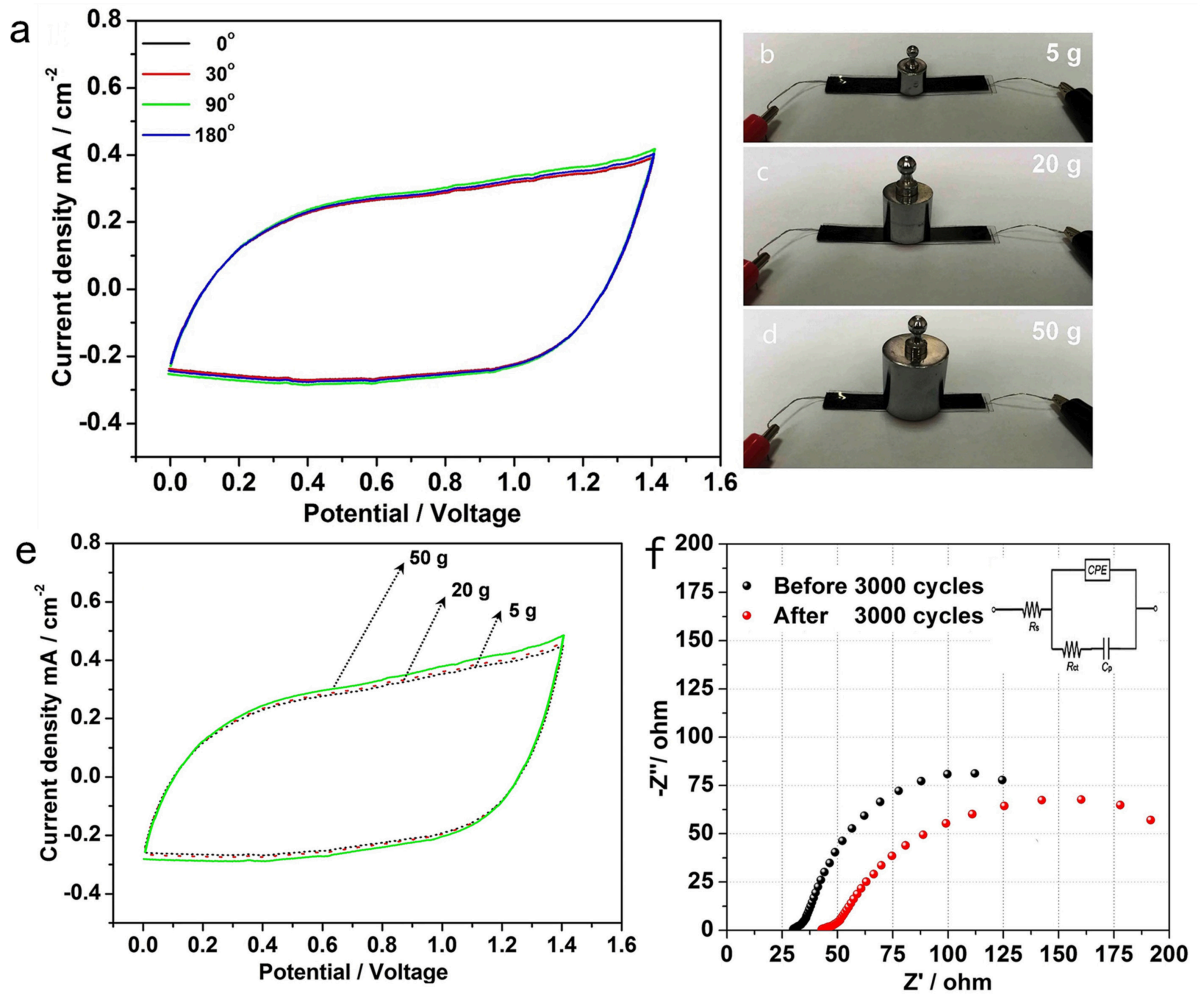
**(a)** CV curves within 0–1.4 V range at scan rate of 5 mV s^−1^ with different bending degrees. The optical photos of the device under different weights **(b)** 5 g; **(c)** 20 g; **(d)** 50 g, and corresponding **(e)** CV curves within 0–1.4 V range at scan rate of 10 mV s^−1^ when under different weights. **(f)** The Nyquist plots of the hybrid supercapacitor before and after 3,000 cycles at a current density of 5.0 mA cm^−2^, inset shows the EIS equivalent circuitry.

To evaluate the potential of the all-solid-state hybrid supercapacitor for flexible energy storage under real conditions, the CV curves of the device at 5 mV s^−1^ were collected under normal and bent conditions. As shown in Figure 4a, when the hybrid supercapacitor was bent to 30°, 90°, 180°, the curves change slightly, suggesting the good capacitance stability of this flexible supercapacitor (Qin et al., 2018; Wang W. et al., 2018). Figures 4b–e show the all-solid-state hybrid supercapacitor under different weights and corresponding CV curves with 0–1.4 V range at a scan rate of 10 mV s^−1^. Similar to the device with different bending angles, the CV curves of the device under different weight (5, 20, and 50 g) change slightly, and the corresponding specific capacitance of the device is well-maintained. All the above results show that this hybrid supercapacitor has excellent mechanical flexibility (Qin et al., 2018; Wang W. et al., 2018).

We test the electrochemical impedance spectra (EIS) of the supercapacitor before and after 3,000 cycles at a current density of 5.0 mA cm^−2^. An equivalent circuit was given in the inset of Figure 4f, which is similar to the circuit employed for the working electrode of a supercapacitor. The EIS data can be fitted by a bulk solution resistance *R*_s_, a charge-transfer resistance *R*_ct_ and a pseudocapacitive element *C*_p_ from the redox process of electrode materials, and a CPE to account for the double-layer capacitance (Pang et al., 2013). As shown in Figure 4f, the intrinsic resistance *R*_s_ of the device before and after 3,000 cycles are around 27.2 and 38.1 Ω, respectively. And the *R*_ct_ after 3,000 cycles is around 218 ohms, which is higher than the 176 ohms of the initial *R*_ct_. The increase of charge transfer resistance may be due to the irreversible reaction between the electrodes and the electrolyte, which is consistent with the decrease in the capacitance after cycling (Figure 3D).

## Conclusion

In summary, a flexible all-solid-state NH_4_NiPO_4_·H_2_O//graphene device was successfully assembled, which showed great performance. When cycled for 3,000 cycle at the current density of 5.0 mA cm^−2^, the hybrid supercapacitor shows 88.8% in capacitance retention rate. The device also showed excellent flexibility, especially when bent to various degrees and under different weights. The as-prepared flexible all-solid-state device could be integrated in to large scale flexible systems that require an energy storage unit. And further study will be focused on improving the device performance.

## Author Contributions

YL, WZ, and HP conceived and designed the experiments. YL, XZ, FW, and KY performed the experiments. XZ, HW, and FR analyzed the data. YL and XZ wrote the paper. HP and WZ revised the paper, which could be found in the list of corrections we have submitted.

### Conflict of Interest Statement

The authors declare that the research was conducted in the absence of any commercial or financial relationships that could be construed as a potential conflict of interest.
