# Supplementary material for: Mesoporous NH4NiPO4·H2O for High-Performance Flexible All-Solid-State Asymmetric Supercapacitors
Source: Front Chem. 2019 Mar 7;7:118. doi: 10.3389/fchem.2019.00118 (PMC6423919; doi:10.3389/fchem.2019.00118)
Supplement: Supplementary file 1 [file Data_Sheet_1.doc]

**Mesoporous NH_4_NiPO_4_·H_2_O for High-performance Flexible All-Solid-State Asymmetric Supercapacitors**

*Yong Liu^1,3*^*†*, Xiaoliang Zhai^1^*†*, Keke Yang^1^*†*, Fei Wang^1^, Huijie Wei^1^, Wanhong Zhang^1*^, Fengzhang Ren^1^, Huan Pang^2^*

1. Collaborative Innovation Center of Nonferrous Metals of Henan Province, Henan Key Laboratory of High-temperature Structural and Functional Materials, School of Materials Science and Engineering, Henan University of Science and Technology, Luoyang 471023, P. R. China

2. School of Chemistry and Chemical Engineering, Yangzhou University, Yangzhou 225002, P. R. China

3. Henan Key Laboratory of Non-ferrous Materials Science & Processing Technology, Henan University of Science and Technology, Luoyang 471023, P. R. China

† These authors contributed equally contribution to this work

***Corresponding author:**

Prof. Yong Liu, [liuyong209@haust.edu.cn](mailto:huanpangchem@hotmail.com)

Prof. Wanhong Zhang, zhangwh@haust.edu.cn


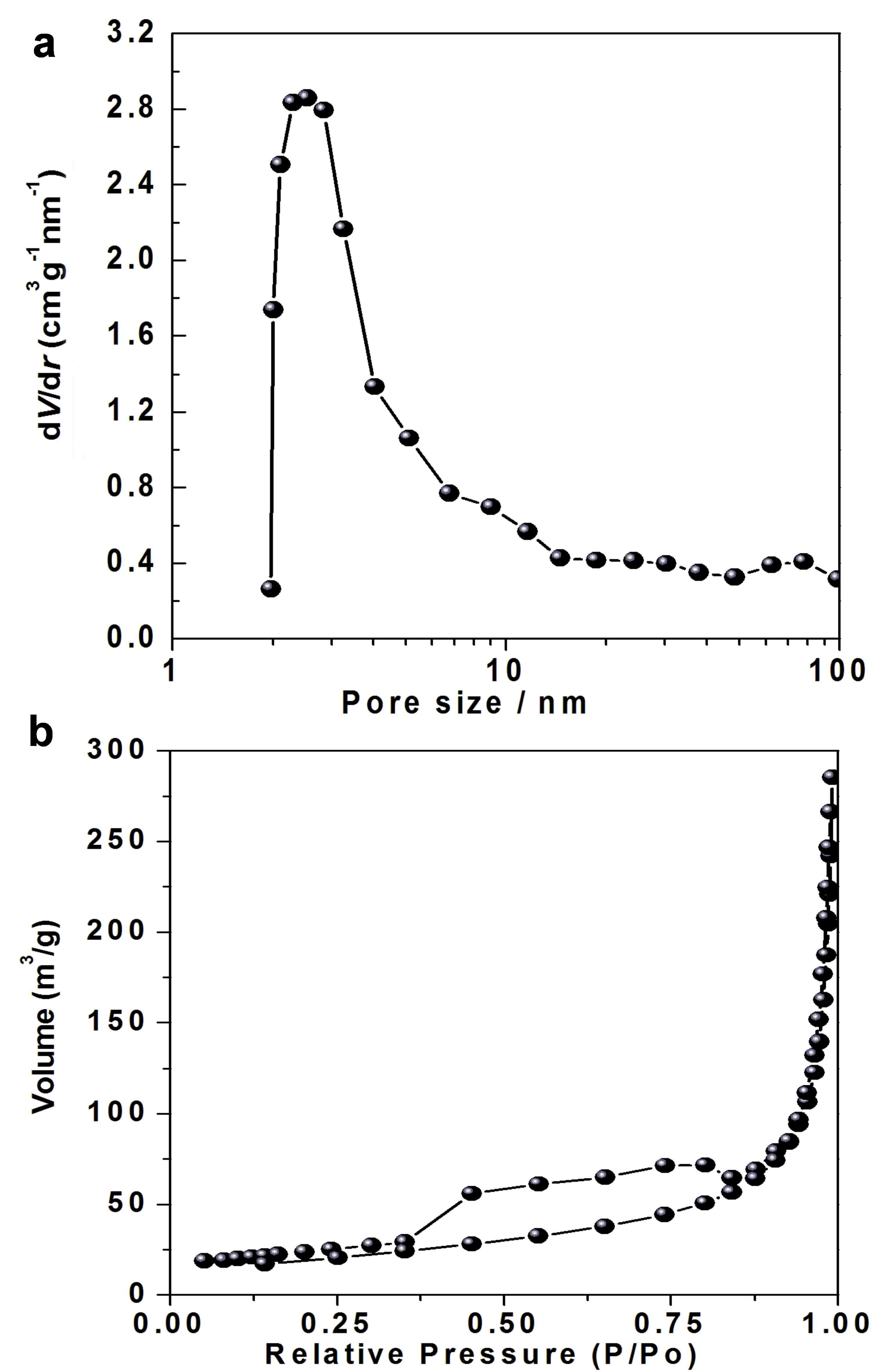


**Fig. S1** (a) The pore-size distribution curve and (b) typical N_2_ adsorption-desorption isotherms of mesoporous NH_4_NiPO_4_·H_2_O.


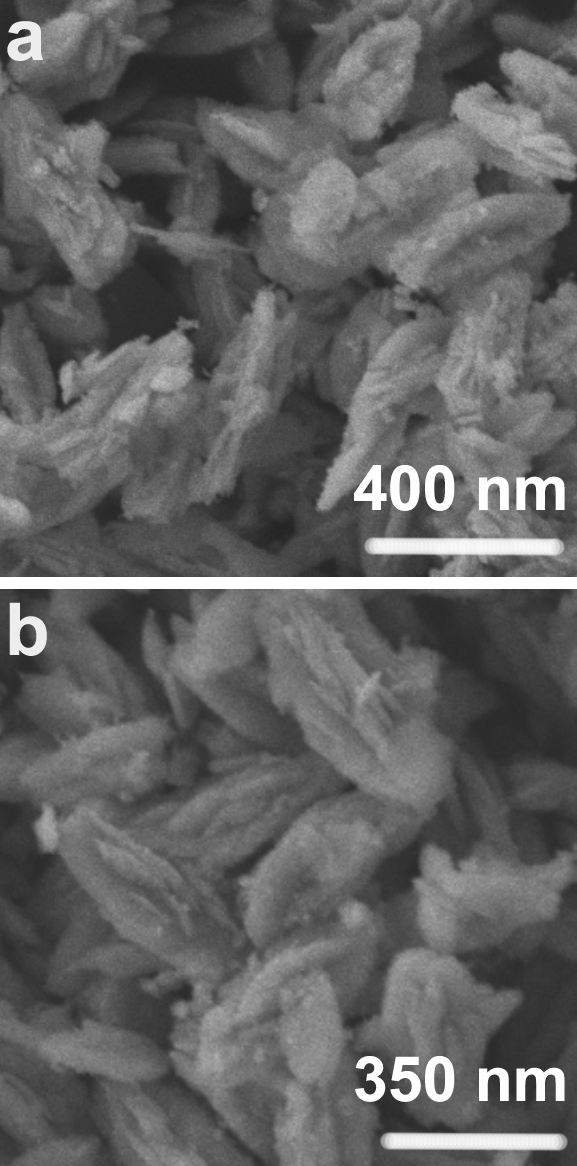


**Fig. S2** (a, b) The SEM images of NH_4_NiPO_4_·H_2_O electrode after 3000 cycles

Table S1 The comparison of specific capacity of NH_4_NiPO_4_·H_2_O//graphene with other supercapacitors

| Electrode Material | Capacitance | References |
| --- | --- | --- |
| MWCNT/PANI | 44.13 mF cm^‐2^ at 0.2 mA cm^‐2^ | (Li et al., 2017) |
| MnO_2_-polypyrrole | 25.9 and 20.3 mF cm^-2^ at 0.2 and 0.5 mA cm^-2^ | (Wang et al., 2014) |
| ZnS@β-Ni(OH)_2_ | 170.97 mF cm^-2^ at 2.0 mA cm^-2^, | (Wei et al., 2016) |
| Co_3_O_4_ | 4.8 mF cm^-2^ at 3 mA cm^-2^ | (Padmanathan et al., 2015) |
| NH_4_NiPO_4_•H_2_O | 180 and 121 mF cm^-2^ at 0.5 and 5 mA cm^-2^ | This work |

Reference

Li, L., Lou, Z., Han, W., Chen, D., Jiang, K., and Shen, G. (2017). Highly Stretchable Micro-Supercapacitor Arrays with Hybrid MWCNT/PANI Electrodes. *Advanced Materials Technologies* 2(3). doi: 10.1002/admt.201600282.

Padmanathan, N., Selladurai, S., and Razeeb, K.M. (2015). Ultra-fast rate capability of a symmetric supercapacitor with a hierarchical Co3O4 nanowire/nanoflower hybrid structure in non-aqueous electrolyte. Rsc Advances 5(17), 12700-12709. doi: 10.1039/c4ra13327g.

Wang, C., Zhan, Y., Wu, L., Li, Y., and Liu, J. (2014). High-voltage and high-rate symmetric supercapacitor based on MnO2-polypyrrole hybrid nanofilm. *Nanotechnology* 25(30). doi: 10.1088/0957-4484/25/30/305401.

Wei, C., Cheng, C., Du, W., Ren, J., Li, M., Dong, J., et al. (2016). Facile synthesis of mesoporous hierarchical ZnS@beta-Ni(OH)(2) microspheres for flexible solid state hybrid supercapacitors. *Rsc Advances* 6(103)**,** 101016-101022. doi: 10.1039/c6ra23549b.
